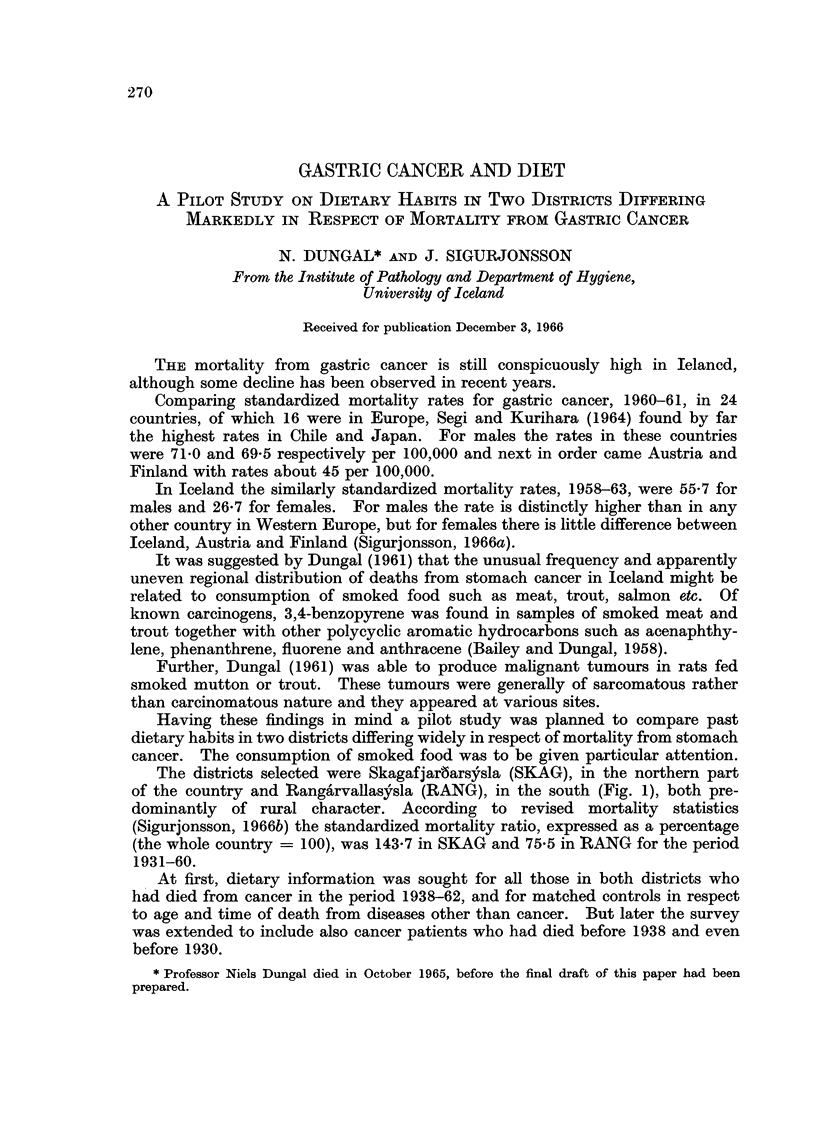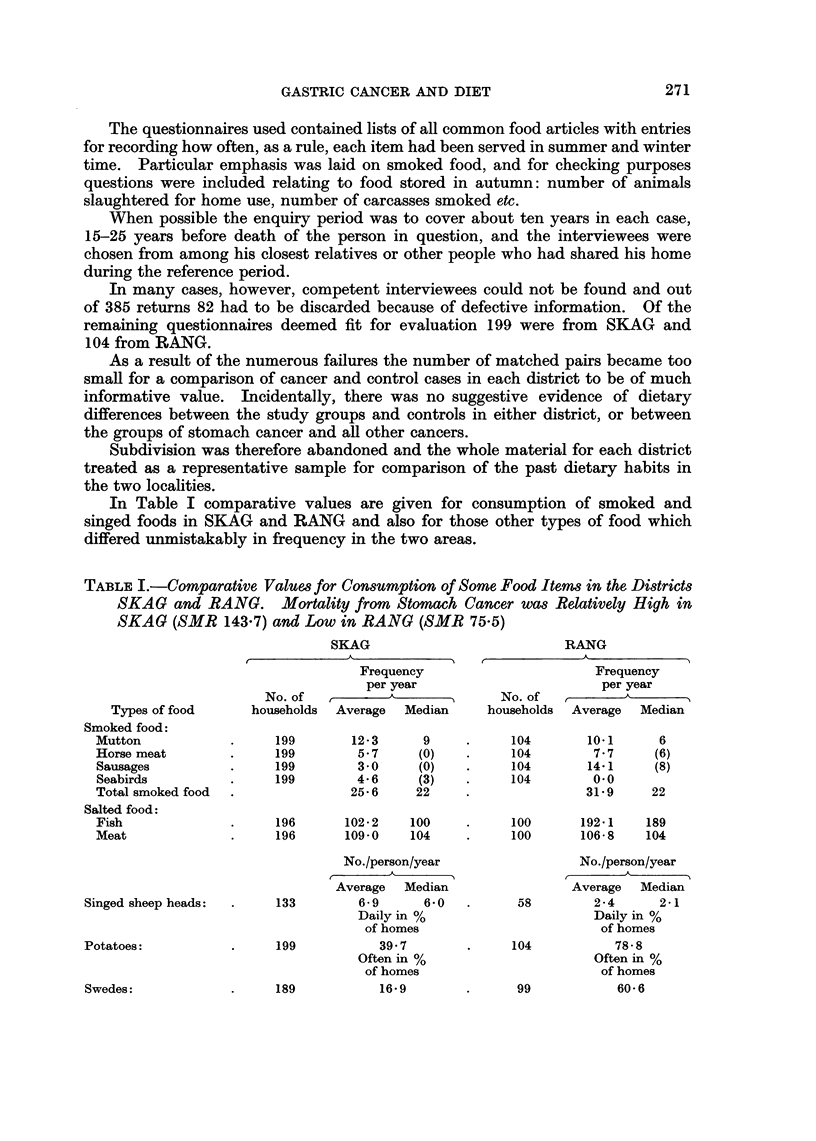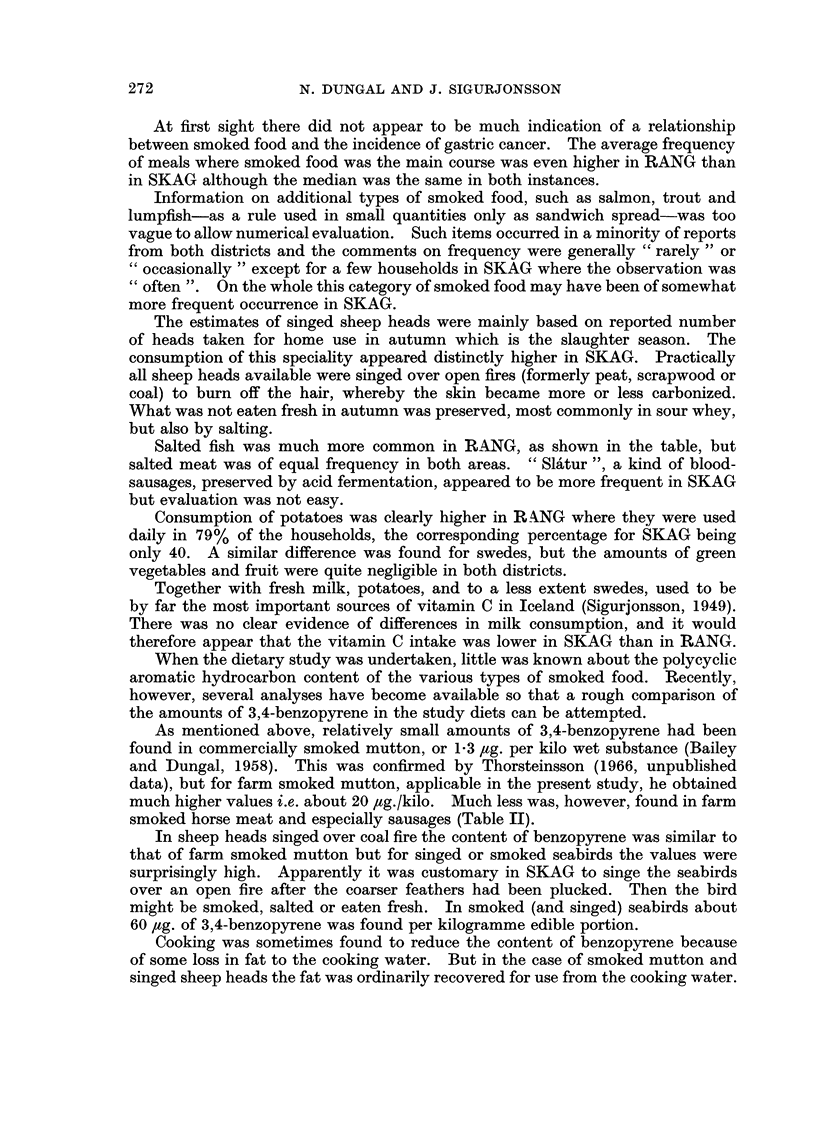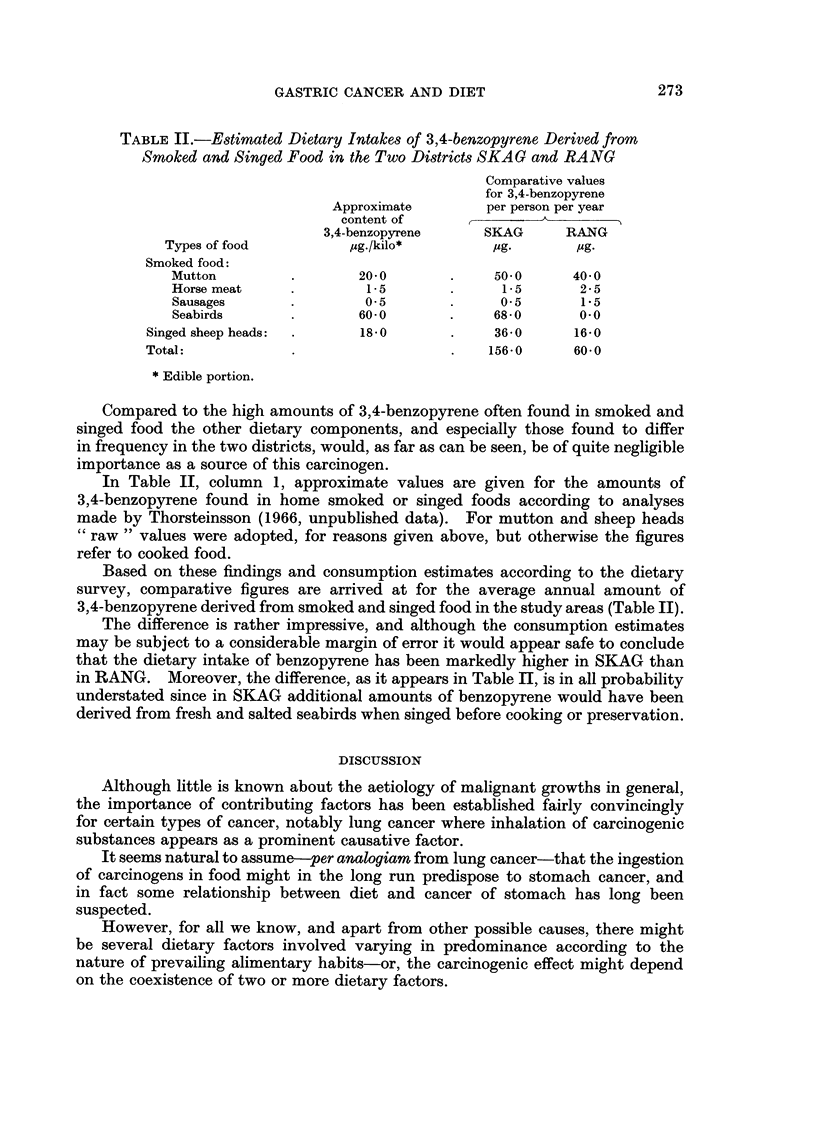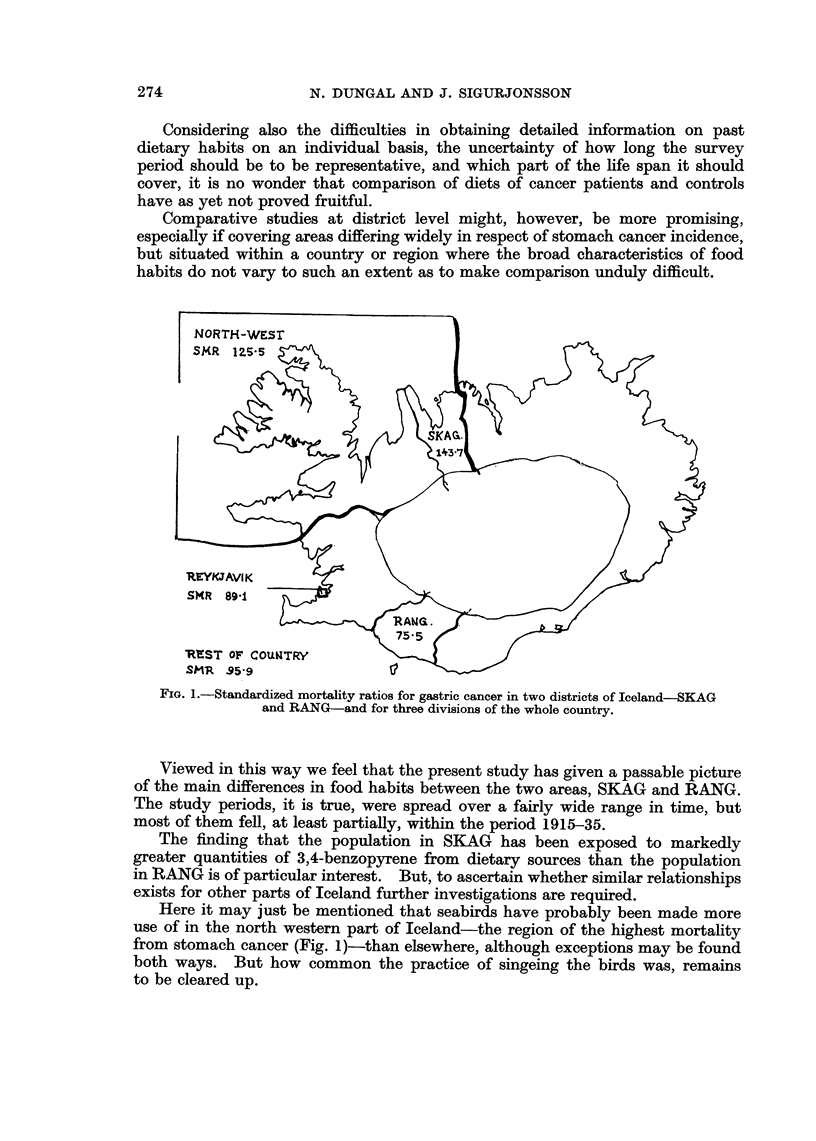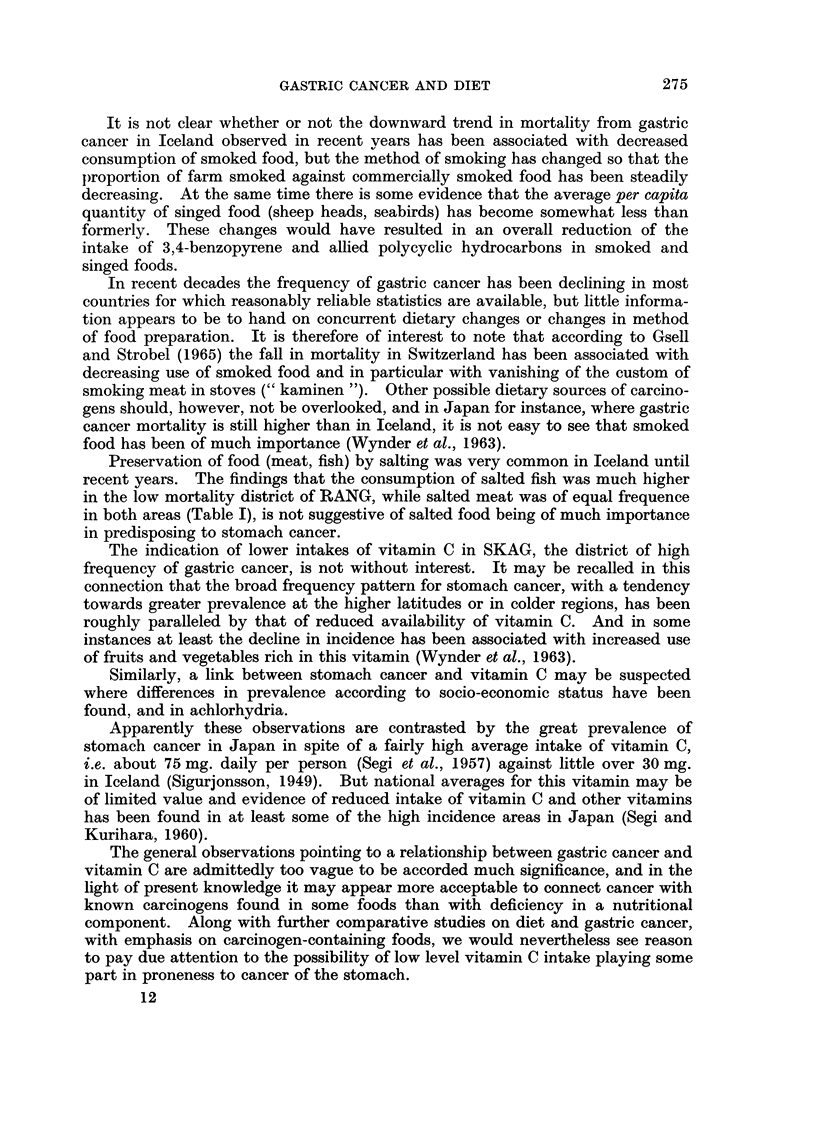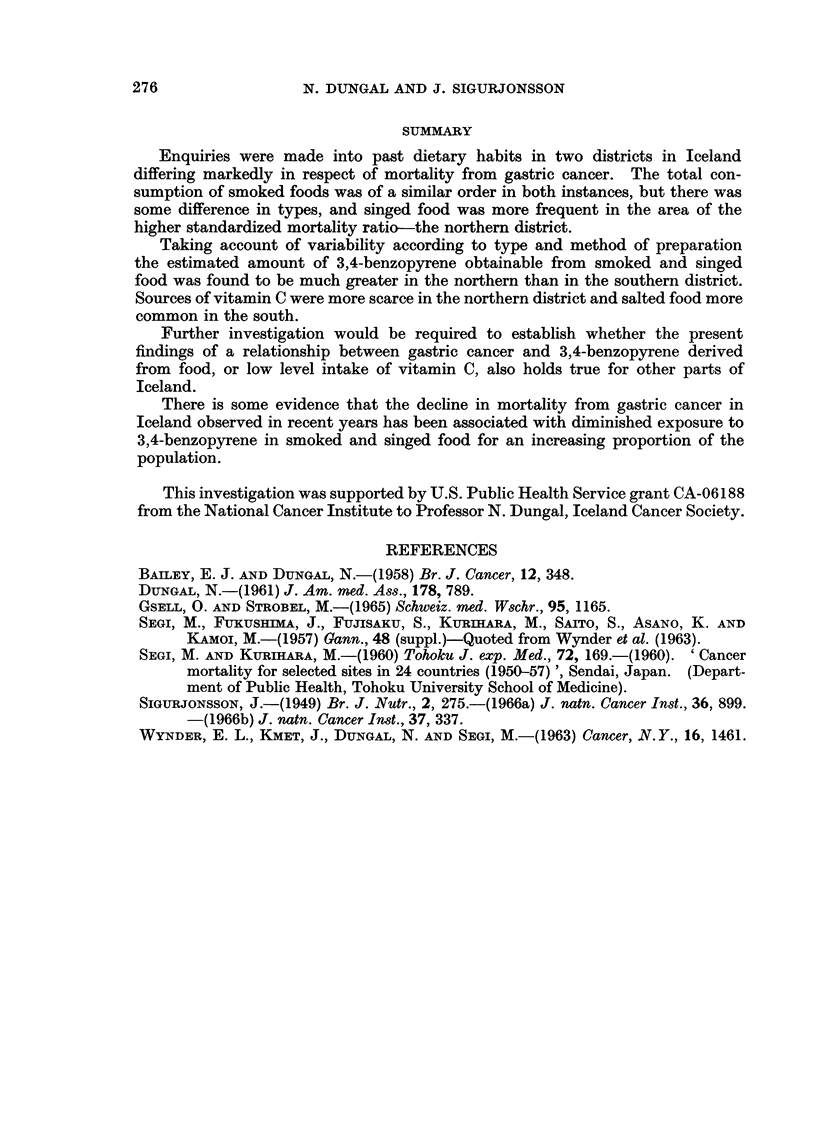# Gastric cancer and diet. A pilot study on dietary habits in two districts differing markedly in respect of mortality from gastric cancer.

**DOI:** 10.1038/bjc.1967.29

**Published:** 1967-06

**Authors:** N. Dungal, J. Sigurjonsson


### GASTRIC CANCER AND DIET

### A Pilot Study on Dietary Habits in Two Districts Differing Markedly in Respect of Mortality from Gastric Cancer

N. DUNGAL* AND J. SIGURJONSSON

*From the Institute of Pathology and Department of Hygiene, University of Iceland*

Received for publication December 3, 1966

THE mortality from gastric cancer is still conspicuously high in Ielancd, although some decline has been observed in recent years.

Comparing standardized mortality rates for gastric cancer, 1960–61, in 24 countries, of which 16 were in Europe, Segi and Kurihara (1964) found by far the highest rates in Chile and Japan. For males the rates in these countries were 71·0 and 69·5 respectively per 100,000 and next in order came Austria and Finland with rates about 45 per 100,000.

In Iceland the similarly standardized mortality rates, 1958–63, were 55·7 for males and 26·7 for females. For males the rate is distinctly higher than in any other country in Western Europe, but for females there is little difference between Iceland, Austria and Finland (Sigurjonsson, 1966*a*).

It was suggested by Dungal (1961) that the unusual frequency and apparently uneven regional distribution of deaths from stomach cancer in Iceland might be related to consumption of smoked food such as meat, trout, salmon *etc.* Of known carcinogens, 3,4-benzopyrene was found in samples of smoked meat and trout together with other polycyclic aromatic hydrocarbons such as acenaphthylene, phenanthrene, fluorene and anthracene (Bailey and Dungal, 1958).

Further, Dungal (1961) was able to produce malignant tumours in rats fed smoked mutton or trout. These tumours were generally of sarcomatous rather than carcinomatous nature and they appeared at various sites.

Having these findings in mind a pilot study was planned to compare past dietary habits in two districts differing widely in respect of mortality from stomach cancer. The consumption of smoked food was to be given particular attention.

The districts selected were Skagafjarðarsýsla (SKAG), in the northern part of the country and Rangárvallasýsla (RANG), in the south (Fig. 1), both predominantly of rural character. According to revised mortality statistics (Sigurjonsson, 1966*b*) the standardized mortality ratio, expressed as a percentage (the whole country = 100), was 143·7 in SKAG and 75·5 in RANG for the period 1931–60.

At first, dietary information was sought for all those in both districts who had died from cancer in the period 1938–62, and for matched controls in respect to age and time of death from diseases other than cancer. But later the survey was extended to include also cancer patients who had died before 1938 and even before 1930.

* Professor Niels Dungal died in October 1965, before the final draft of this paper had been prepared.

The questionnaires used contained lists of all common food articles with entries for recording how often, as a rule, each item had been served in summer and winter time. Particular emphasis was laid on smoked food, and for checking purposes questions were included relating to food stored in autumn: number of animals slaughtered for home use, number of carcasses smoked *etc.*

When possible the enquiry period was to cover about ten years in each case, 15–25 years before death of the person in question, and the interviewees were chosen from among his closest relatives or other people who had shared his home during the reference period.

In many cases, however, competent interviewees could not be found and out of 385 returns 82 had to be discarded because of defective information. Of the remaining questionnaires deemed fit for evaluation 199 were from SKAG and 104 from RANG.

As a result of the numerous failures the number of matched pairs became too small for a comparison of cancer and control cases in each district to be of much informative value. Incidentally, there was no suggestive evidence of dietary differences between the study groups and controls in either district, or between the groups of stomach cancer and all other cancers.

Subdivision was therefore abandoned and the whole material for each district treated as a representative sample for comparison of the past dietary habits in the two localities.

In Table I comparative values are given for consumption of smoked and singed foods in SKAG and RANG and also for those other types of food which differed unmistakably in frequency in the two areas.

TABLE I.—*Comparative Values for Consumption of Some Food Items in the Districts SKAG and RANG. Mortality from Stomach Cancer was Relatively High in SKAG (SMR* 143·7) *and Low in RANG (SMR* 75·5)

| | SKAG | | | RANG | | |
|---|---|---|---|---|---|---|
| | | Frequency per year | | | Frequency per year | |
| Types of food | No. of households | Average | Median | No. of households | Average | Median |
| Smoked food: | | | | | | |
| Mutton | 199 | 12·3 | 9 | 104 | 10·1 | 6 |
| Horse meat | 199 | 5·7 | (0) | 104 | 7·7 | (6) |
| Sausages | 199 | 3·0 | (0) | 104 | 14·1 | (8) |
| Seabirds | 199 | 4·6 | (3) | 104 | 0·0 | |
| Total smoked food | | 25·6 | 22 | | 31·9 | 22 |
| Salted food: | | | | | | |
| Fish | 196 | 102·2 | 100 | 100 | 192·1 | 189 |
| Meat | 196 | 109·0 | 104 | 100 | 106·8 | 104 |
| | | No./person/year | | | No./person/year | |
| | | Average | Median | | Average | Median |
| Singed sheep heads: | 133 | 6·9 | 6·0 | 58 | 2·4 | 2·1 |
| | | Daily in % of homes | | | Daily in % of homes | |
| Potatoes: | 199 | 39·7 | | 104 | 78·8 | |
| | | Often in % of homes | | | Often in % of homes | |
| Swedes: | 189 | 16·9 | | 99 | 60·6 | |

At first sight there did not appear to be much indication of a relationship between smoked food and the incidence of gastric cancer. The average frequency of meals where smoked food was the main course was even higher in RANG than in SKAG although the median was the same in both instances.

Information on additional types of smoked food, such as salmon, trout and lumpfish—as a rule used in small quantities only as sandwich spread—was too vague to allow numerical evaluation. Such items occurred in a minority of reports from both districts and the comments on frequency were generally " rarely " or " occasionally " except for a few households in SKAG where the observation was " often ". On the whole this category of smoked food may have been of somewhat more frequent occurrence in SKAG.

The estimates of singed sheep heads were mainly based on reported number of heads taken for home use in autumn which is the slaughter season. The consumption of this speciality appeared distinctly higher in SKAG. Practically all sheep heads available were singed over open fires (formerly peat, scrapwood or coal) to burn off the hair, whereby the skin became more or less carbonized. What was not eaten fresh in autumn was preserved, most commonly in sour whey, but also by salting.

Salted fish was much more common in RANG, as shown in the table, but salted meat was of equal frequency in both areas. " Slátur ", a kind of blood-sausages, preserved by acid fermentation, appeared to be more frequent in SKAG but evaluation was not easy.

Consumption of potatoes was clearly higher in RANG where they were used daily in 79% of the households, the corresponding percentage for SKAG being only 40. A similar difference was found for swedes, but the amounts of green vegetables and fruit were quite negligible in both districts.

Together with fresh milk, potatoes, and to a less extent swedes, used to be by far the most important sources of vitamin C in Iceland (Sigurjonsson, 1949). There was no clear evidence of differences in milk consumption, and it would therefore appear that the vitamin C intake was lower in SKAG than in RANG.

When the dietary study was undertaken, little was known about the polycyclic aromatic hydrocarbon content of the various types of smoked food. Recently, however, several analyses have become available so that a rough comparison of the amounts of 3,4-benzopyrene in the study diets can be attempted.

As mentioned above, relatively small amounts of 3,4-benzopyrene had been found in commercially smoked mutton, or 1·3 $\mu$g. per kilo wet substance (Bailey and Dungal, 1958). This was confirmed by Thorsteinsson (1966, unpublished data), but for farm smoked mutton, applicable in the present study, he obtained much higher values *i.e.* about 20 $\mu$g./kilo. Much less was, however, found in farm smoked horse meat and especially sausages (Table II).

In sheep heads singed over coal fire the content of benzopyrene was similar to that of farm smoked mutton but for singed or smoked seabirds the values were surprisingly high. Apparently it was customary in SKAG to singe the seabirds over an open fire after the coarser feathers had been plucked. Then the bird might be smoked, salted or eaten fresh. In smoked (and singed) seabirds about 60 $\mu$g. of 3,4-benzopyrene was found per kilogramme edible portion.

Cooking was sometimes found to reduce the content of benzopyrene because of some loss in fat to the cooking water. But in the case of smoked mutton and singed sheep heads the fat was ordinarily recovered for use from the cooking water.

TABLE II.—*Estimated Dietary Intakes of* 3,4-*benzopyrene Derived from Smoked and Singed Food in the Two Districts SKAG and RANG*

| Types of food | Approximate content of 3,4-benzopyrene μg./kilo* | Comparative values for 3,4-benzopyrene per person per year SKAG μg. | RANG μg. |
|---|---|---|---|
| Smoked food: | | | |
| Mutton | 20·0 | 50·0 | 40·0 |
| Horse meat | 1·5 | 1·5 | 2·5 |
| Sausages | 0·5 | 0·5 | 1·5 |
| Seabirds | 60·0 | 68·0 | 0·0 |
| Singed sheep heads: | 18·0 | 36·0 | 16·0 |
| Total: | | 156·0 | 60·0 |

* Edible portion.

Compared to the high amounts of 3,4-benzopyrene often found in smoked and singed food the other dietary components, and especially those found to differ in frequency in the two districts, would, as far as can be seen, be of quite negligible importance as a source of this carcinogen.

In Table II, column 1, approximate values are given for the amounts of 3,4-benzopyrene found in home smoked or singed foods according to analyses made by Thorsteinsson (1966, unpublished data). For mutton and sheep heads " raw " values were adopted, for reasons given above, but otherwise the figures refer to cooked food.

Based on these findings and consumption estimates according to the dietary survey, comparative figures are arrived at for the average annual amount of 3,4-benzopyrene derived from smoked and singed food in the study areas (Table II).

The difference is rather impressive, and although the consumption estimates may be subject to a considerable margin of error it would appear safe to conclude that the dietary intake of benzopyrene has been markedly higher in SKAG than in RANG. Moreover, the difference, as it appears in Table II, is in all probability understated since in SKAG additional amounts of benzopyrene would have been derived from fresh and salted seabirds when singed before cooking or preservation.

### DISCUSSION

Although little is known about the aetiology of malignant growths in general, the importance of contributing factors has been established fairly convincingly for certain types of cancer, notably lung cancer where inhalation of carcinogenic substances appears as a prominent causative factor.

It seems natural to assume—*per analogiam* from lung cancer—that the ingestion of carcinogens in food might in the long run predispose to stomach cancer, and in fact some relationship between diet and cancer of stomach has long been suspected.

However, for all we know, and apart from other possible causes, there might be several dietary factors involved varying in predominance according to the nature of prevailing alimentary habits—or, the carcinogenic effect might depend on the coexistence of two or more dietary factors.

Considering also the difficulties in obtaining detailed information on past dietary habits on an individual basis, the uncertainty of how long the survey period should be to be representative, and which part of the life span it should cover, it is no wonder that comparison of diets of cancer patients and controls have as yet not proved fruitful.

Comparative studies at district level might, however, be more promising, especially if covering areas differing widely in respect of stomach cancer incidence, but situated within a country or region where the broad characteristics of food habits do not vary to such an extent as to make comparison unduly difficult.

FIG. 1.—Standardized mortality ratios for gastric cancer in two districts of Iceland—SKAG and RANG—and for three divisions of the whole country.

Viewed in this way we feel that the present study has given a passable picture of the main differences in food habits between the two areas, SKAG and RANG. The study periods, it is true, were spread over a fairly wide range in time, but most of them fell, at least partially, within the period 1915–35.

The finding that the population in SKAG has been exposed to markedly greater quantities of 3,4-benzopyrene from dietary sources than the population in RANG is of particular interest. But, to ascertain whether similar relationships exists for other parts of Iceland further investigations are required.

Here it may just be mentioned that seabirds have probably been made more use of in the north western part of Iceland—the region of the highest mortality from stomach cancer (Fig. 1)—than elsewhere, although exceptions may be found both ways. But how common the practice of singeing the birds was, remains to be cleared up.

It is not clear whether or not the downward trend in mortality from gastric cancer in Iceland observed in recent years has been associated with decreased consumption of smoked food, but the method of smoking has changed so that the proportion of farm smoked against commercially smoked food has been steadily decreasing. At the same time there is some evidence that the average *per capita* quantity of singed food (sheep heads, seabirds) has become somewhat less than formerly. These changes would have resulted in an overall reduction of the intake of 3,4-benzopyrene and allied polycyclic hydrocarbons in smoked and singed foods.

In recent decades the frequency of gastric cancer has been declining in most countries for which reasonably reliable statistics are available, but little information appears to be to hand on concurrent dietary changes or changes in method of food preparation. It is therefore of interest to note that according to Gsell and Strobel (1965) the fall in mortality in Switzerland has been associated with decreasing use of smoked food and in particular with vanishing of the custom of smoking meat in stoves (" kaminen "). Other possible dietary sources of carcinogens should, however, not be overlooked, and in Japan for instance, where gastric cancer mortality is still higher than in Iceland, it is not easy to see that smoked food has been of much importance (Wynder *et al.*, 1963).

Preservation of food (meat, fish) by salting was very common in Iceland until recent years. The findings that the consumption of salted fish was much higher in the low mortality district of RANG, while salted meat was of equal frequence in both areas (Table I), is not suggestive of salted food being of much importance in predisposing to stomach cancer.

The indication of lower intakes of vitamin C in SKAG, the district of high frequency of gastric cancer, is not without interest. It may be recalled in this connection that the broad frequency pattern for stomach cancer, with a tendency towards greater prevalence at the higher latitudes or in colder regions, has been roughly paralleled by that of reduced availability of vitamin C. And in some instances at least the decline in incidence has been associated with increased use of fruits and vegetables rich in this vitamin (Wynder *et al.*, 1963).

Similarly, a link between stomach cancer and vitamin C may be suspected where differences in prevalence according to socio-economic status have been found, and in achlorhydria.

Apparently these observations are contrasted by the great prevalence of stomach cancer in Japan in spite of a fairly high average intake of vitamin C, *i.e.* about 75 mg. daily per person (Segi *et al.*, 1957) against little over 30 mg. in Iceland (Sigurjonsson, 1949). But national averages for this vitamin may be of limited value and evidence of reduced intake of vitamin C and other vitamins has been found in at least some of the high incidence areas in Japan (Segi and Kurihara, 1960).

The general observations pointing to a relationship between gastric cancer and vitamin C are admittedly too vague to be accorded much significance, and in the light of present knowledge it may appear more acceptable to connect cancer with known carcinogens found in some foods than with deficiency in a nutritional component. Along with further comparative studies on diet and gastric cancer, with emphasis on carcinogen-containing foods, we would nevertheless see reason to pay due attention to the possibility of low level vitamin C intake playing some part in proneness to cancer of the stomach.

SUMMARY

Enquiries were made into past dietary habits in two districts in Iceland differing markedly in respect of mortality from gastric cancer. The total consumption of smoked foods was of a similar order in both instances, but there was some difference in types, and singed food was more frequent in the area of the higher standardized mortality ratio—the northern district.

Taking account of variability according to type and method of preparation the estimated amount of 3,4-benzopyrene obtainable from smoked and singed food was found to be much greater in the northern than in the southern district. Sources of vitamin C were more scarce in the northern district and salted food more common in the south.

Further investigation would be required to establish whether the present findings of a relationship between gastric cancer and 3,4-benzopyrene derived from food, or low level intake of vitamin C, also holds true for other parts of Iceland.

There is some evidence that the decline in mortality from gastric cancer in Iceland observed in recent years has been associated with diminished exposure to 3,4-benzopyrene in smoked and singed food for an increasing proportion of the population.

This investigation was supported by U.S. Public Health Service grant CA-06188 from the National Cancer Institute to Professor N. Dungal, Iceland Cancer Society.

REFERENCES

BAILEY, E. J. AND DUNGAL, N.—(1958) *Br. J. Cancer*, **12**, 348.

DUNGAL, N.—(1961) *J. Am. med. Ass.*, **178**, 789.

GSELL, O. AND STROBEL, M.—(1965) *Schweiz. med. Wschr.*, **95**, 1165.

SEGI, M., FUKUSHIMA, J., FUJISAKU, S., KURIHARA, M., SAITO, S., ASANO, K. AND KAMOI, M.—(1957) *Gann.*, **48** (suppl.)—Quoted from Wynder *et al.* (1963).

SEGI, M. AND KURIHARA, M.—(1960) *Tohoku J. exp. Med.*, **72**, 169.—(1960). ' Cancer mortality for selected sites in 24 countries (1950–57) ', Sendai, Japan. (Department of Public Health, Tohoku University School of Medicine).

SIGURJONSSON, J.—(1949) *Br. J. Nutr.*, **2**, 275.—(1966a) *J. natn. Cancer Inst.*, **36**, 899. —(1966b) *J. natn. Cancer Inst.*, **37**, 337.

WYNDER, E. L., KMET, J., DUNGAL, N. AND SEGI, M.—(1963) *Cancer, N.Y.*, **16**, 1461.